# IL-24 Promotes Apoptosis through cAMP-Dependent PKA Pathways in Human Breast Cancer Cells

**DOI:** 10.3390/ijms19113561

**Published:** 2018-11-12

**Authors:** Leah Persaud, Jason Mighty, Xuelin Zhong, Ashleigh Francis, Marifer Mendez, Hilal Muharam, Stephen M. Redenti, Dibash Das, Bertal Huseyin Aktas, Moira Sauane

**Affiliations:** 1 Department of Biological Sciences, Herbert H. Lehman College, City University of New York, 250 Bedford Park Boulevard West, Bronx, NY 10468, USA; jason.mighty@lehman.cuny.edu (J.M.); xzhong@gradcenter.cuny.edu (X.Z.); ashleighfrancis64@gmail.com (A.F.); marifer.mendez@lc.cuny.edu (M.M.); hilal.muharam@lc.cuny.edu (H.M.); stephen.redenti@lehman.cuny.edu (S.M.R.); dd791@hunter.cuny.edu (D.D.); 2 Biological Sciences Doctoral Program, The Graduate Center, City University of New York, 365 Fifth Avenue, Room 4315, New York, NY 10016, USA; 3 Department of Medicine, Brigham and Women’s Hospital, 75 Francis Street, Boston, MA 02115, USA; huseyin_aktas@hms.harvard.edu; 4 Harvard Medical School, and Brigham and Women’s Hospital, Division of Hematology, 75 Francis Street, Boston, MA 02115, USA

**Keywords:** interleukin 24, melanoma differentiation associated gene 7, protein kinase A, apoptosis, p53, cytokine, ATF4, extrinsic apoptosis, translation initiation, cancer therapy, gene therapy

## Abstract

Interleukin 24 (IL-24) is a tumor-suppressing protein, which inhibits angiogenesis and induces cancer cell-specific apoptosis. We have shown that IL-24 regulates apoptosis through phosphorylated eukaryotic initiation factor 2 alpha (eIF2α) during endoplasmic reticulum (ER) stress in cancer. Although multiple stresses converge on eIF2α phosphorylation, the cellular outcome is not always the same. In particular, ER stress-induced apoptosis is primarily regulated through the extent of eIF2α phosphorylation and activating transcription factor 4 (ATF4) action. Our studies show for the first time that cyclic adenosine monophosphate (cAMP)-dependent protein kinase A (PKA) activation is required for IL-24-induced cell death in a variety of breast cancer cell lines and this event increases ATF4 activity. We demonstrate an undocumented role for PKA in regulating IL-24-induced cell death, whereby PKA stimulates phosphorylation of p38 mitogen-activated protein kinase and upregulates extrinsic apoptotic factors of the Fas/FasL signaling pathway and death receptor 4 expression. We also demonstrate that phosphorylation and nuclear import of tumor suppressor TP53 occurs downstream of IL-24-mediated PKA activation. These discoveries provide the first mechanistic insights into the function of PKA as a key regulator of the extrinsic pathway, ER stress, and TP53 activation triggered by IL-24.

## 1. Introduction

Interleukin-24 (IL-24) is a member of the IL-10 protein family and it displays broad cancer-specific suppressor effects [1,2,3,4,5,6,7,8]. Notably, clinical and pre-clinical studies have indicated that IL-24 displays prominent antitumor action [9]. The tumor suppressor activities of IL-24 include the inhibition of angiogenesis, invasion, and metastasis, sensitization to chemotherapy, and the induction of cancer-specific apoptosis [1,2,3,4,5,6,7,8]. Given its ubiquitous apoptotic effect on malignant cells, the lack of an effect on normal cells, and the absence of significant side effects, IL-24 is an important candidate for cancer therapy (Reviewed in [8]). 

We have shown that overexpression of IL-24 is implicated in endoplasmic reticulum (ER) stress-mediated apoptosis in cancer cells [10]. We have recently demonstrated that IL-24 activates protein kinase R (PKR)-like endoplasmic reticulum kinase (PERK), one of three canonical ER-stress response pathways. PERK phosphorylates the alpha subunit of the eukaryotic translation initiation factor 2 (eIF2a) which inhibits translation initiation while inducing the expression of activating transcription factor 4 (ATF4) and DNA damage-inducible transcript 3, also known as C/EBP homologous (GADD153/CHOP) proteins. Thus, the PERK/eIF2α/ATF4/CHOP axis appears to be essential for the induction of apoptosis by IL-24. Previous studies have shown that protein kinase A (PKA) can also induce ATF4 expression, leading to apoptosis in human liver carcinoma cells after treatment with palmitate, a saturated fatty acid, implicated in ER stress [11]. 

PKA is a serine/threonine kinase that phosphorylates a multitude of proteins in response to fluctuations in cyclic 3′,5′-adenosine monophosphate (cAMP) levels. PKA is a holoenzyme consisting of two catalytic subunits that bind to a dimer of identical regulatory subunits [12]. Under basal conditions, PKA is inactive, but it can become activated when cAMP levels rise in response to various stimuli. Once cAMP binds to the PKA regulatory subunits, PKA catalytic subunits are released as active monomers, which then catalyzes substrate phosphorylation [12]. The cAMP–PKA signaling integrates downstream pathways to regulate numerous cellular responses, including cell proliferation and survival, metabolism, cell cycle regulation, cytoskeleton remodeling, and ion channel regulation (Reviewed in [12]). 

In terms of ER stress and ATF4 activation, after the delayed activation of PKA by palmitate, ATF4 interacts with cAMP-responsive element-binding protein 1 (CREB1), a downstream target of PKA, to bind to the *Atf4* promoter, leading to sustained ATF4 protein expression [11]. It is suggested that this feedback loop involving activated PKA is necessary for the induction of apoptosis via ER-stress and CREB1 phosphorylation. In addition, it has been shown that inhibition of PKA by dihydrochloride (H-89) prevents ATF4 and CHOP induction in cells treated with exendin-4, a glucagon-like peptide 1 receptor agonist [13]. Due to effect of PKA on ATF4, a key target in the ER stress pathway, and the role of PKA as a key growth regulator, we hypothesize that PKA is an upstream mediator of IL-24 killing activity, and that it may regulate several IL-24 downstream signaling pathways, including ATF4 activation. 

In this report, we document for the first time that PKA plays a decisive role in IL-24-mediated apoptosis. These studies define PKA as a key mediator of IL-24 induction of ATF4 activation, extrinsic apoptosis, activator of TP53, and p38 mitogen-activated protein kinase (MAPK). These findings are important in our knowledge of IL-24 as a tumor suppressor protein, as well as an immunomodulatory cytokine.

## 2. Results

### 2.1. IL-24 Regulates the Expression and Phosphorylation of ATF4

We have recently shown that IL-24 inhibits translation initiation by phosphorylating eIF2α during ER stress [14]. Despite this, it is unclear as to why lL-24 induces its apoptotic effect through ER stress mechanisms. ER stress activates both pro-survival and pro-apoptotic pathways; however, a particularly strong or prolonged period of ER stress can overwhelm pro-survival mechanisms, tipping the balance toward apoptotic pathways, and thus preventing tumor development, growth, and invasion. Multiple studies show that different environmental and physiological stresses can affect the duration and level of eIF2α phosphorylation and ATF4 induction and protein interactions, determining cell outcome [15,16,17].

**Figure 1:**
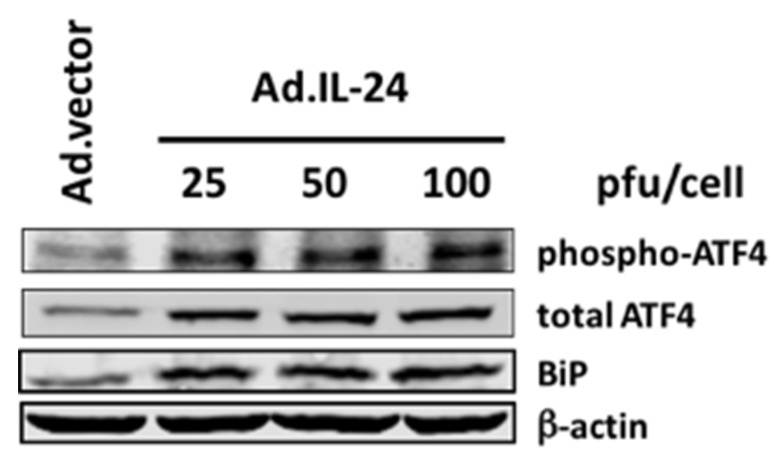
IL-24 activates ATF4 in a dosage dependent manner. MCF-7 cells were treated for 72 h with Ad.IL-24 (25, 50, and 100 plaque-forming units (pfu) per cell) or Ad.vector (100 pfu per cell). Cells were collected, protein purified, and subjected to Western blot analysis to detect phospho-ATF4, total ATF4, BiP, and β-actin.

Therefore, we analyzed whether IL-24 affects the expression of ATF4. We treated MCF-7 human breast cancer cells with increasing concentrations of adenovirus vector expressing IL-24 (Ad.IL-24) for 72 h, and analyzed the expression of ATF4 protein by Western blot. As shown in Figure 1, IL-24 induced both ATF4 expression and ATF4 phosphorylation on serine 245. It has been shown that the phosphorylation of ATF4 at the serine residue 245 upregulates *Atf4* transcriptional activity [18]. We also show that in MCF-7 cells, IL-24 activates binding of immunoglobulin protein (BiP), a downstream marker of ATF4 activation, in a concentration-dependent manner [19]. 

### 2.2. IL-24-Mediated Activation of PKA

**Figure 2:**
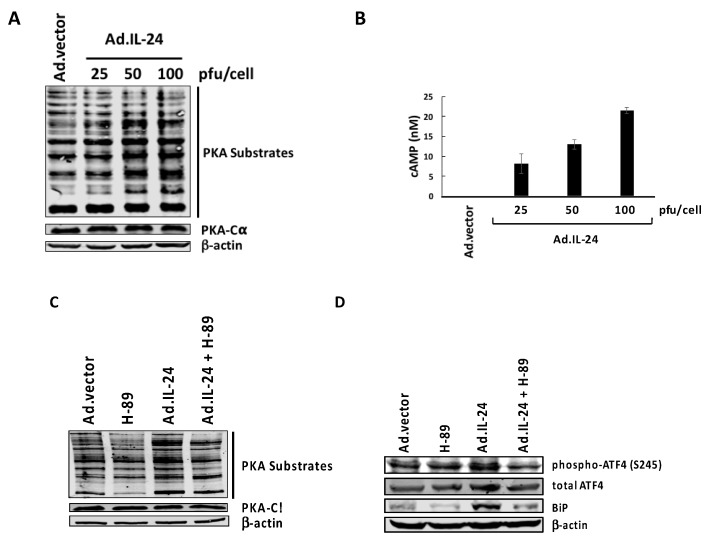
IL-24 activates protein kinase A (PKA) in a concentration-dependent manner. (**A**) MCF-7 cells were treated for 72 h with Ad.IL-24 (25, 50, and 100 pfu per cell) or Ad.vector (100 pfu per cell). Cells were collected, protein purified, and subjected to Western blot analysis to detect phospho-PKA substrates, PKA-Cα subunits, and β-actin proteins. (**B**) MCF-7 cells were treated with Ad.vector (100 pfu/cell) or Ad.IL-24 (25, 50, and 100 pfu per cell) and then assayed for the production of cAMP after 20 h of treatment. Numbers represent mean cyclic adenosine monophosphate (cAMP) (nM) concentration after normalization to control. An average of three independent experiments is shown ± SE (*n* = 9). (**C**,**D**) MCF-7 cells were treated for 72 h with Ad.vector (control) or Ad.IL-24 at 100 pfu/cell, and either untreated or treated with 10 μM H-89 for 72 h. Cells were collected, protein purified, and subjected to Western blot analysis to detect phospho-PKA substrates, PKA-Cα subunit, phospho-ATF4, total ATF4, and β-actin.

ATF4 expression is induced at the translational level, due to eIF2α phosphorylation and at the transcriptional level due to PKA activity [11,14,20]. To determine if IL-24 activates PKA, we examined the profiles of known downstream substrates of PKA which are phosphorylated on PKA-specific serine or threonine residues. We found that with increasing concentrations of IL-24, phosphorylation levels of PKA substrates substantially increased 72 h post-infection in MCF-7 breast cancer cells (Figure 2A). We attribute this increased substrate phosphorylation to PKA activation, rather than an increased expression of PKA, since the protein levels of the PKA catalytic α subunit remained unchanged, despite increasing levels of IL-24 (Figure 2A). This IL-24-mediated activation of PKA is reversed in response to the PKA inhibitor H-89 (Figure 2C). Cyclic 3′,5′-adenosine monophosphate (cAMP) levels, which are a known activator of PKA, also increases in a concentration-dependent manner after treatment with IL-24 (Figure 2B). To determine whether PKA is involved in the activation of ATF4, we used PKA inhibitor, H-89, in conjunction with IL-24 treatment. Figure 2D shows a decrease in ATF4 phosphorylation at serine 245 when IL-24 is overexpressed, and PKA is inhibited demonstrating the involvement of PKA in IL-24 activation of ATF4. 

### 2.3. Inhibiting PKA Activity Abrogates the IL-24 Killing Effect

**Figure 3:**
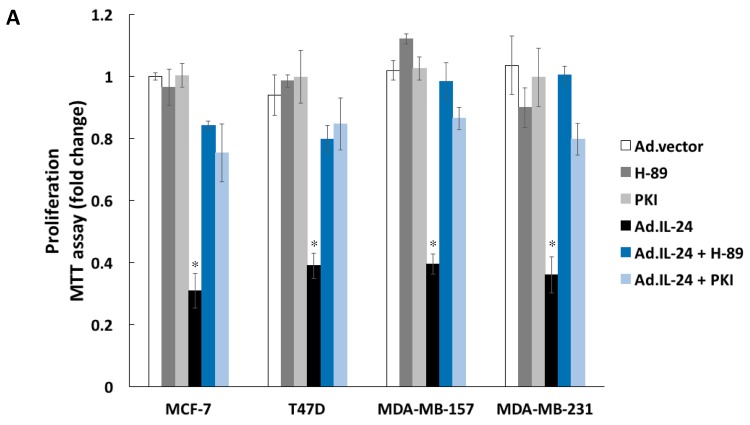

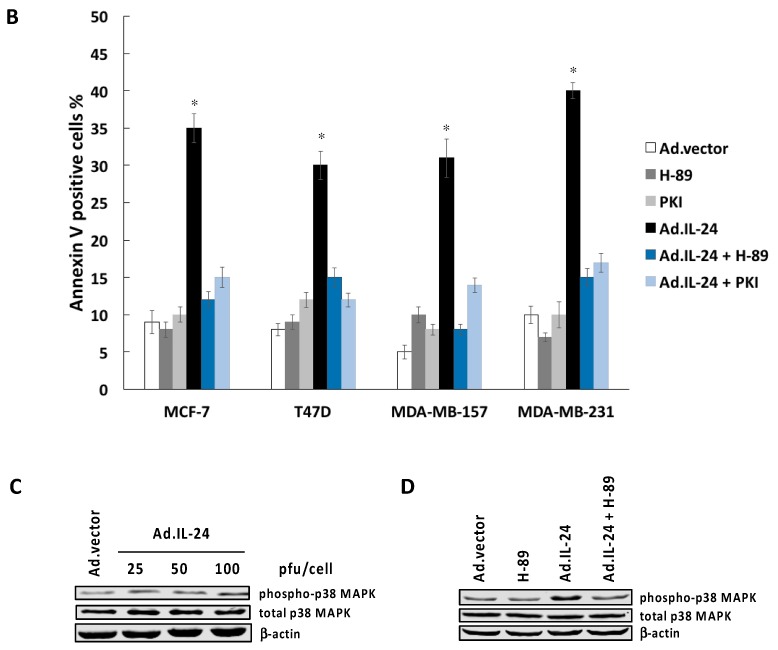
The IL-24 killing effect is decreased in the presence of PKA inhibitors. (**A**) Human breast MCF-7, MDA-MB-231, MDA-MB-157, and T47D cancer cells were incubated with 10 μM of PKA inhibitor, H-89, or PKI, with or without Ad.IL-24 (100 pfu per cell) or Ad.vector (100 pfu per cell), and cell viability was determined by the MTT proliferation assay five days after treatment. Numbers represent the ratio of specific treatments to values in control cells (Ad.vector). An average of three independent experiments is shown ± SD as errors bars. *, *p* < 0.001 comparted to Ad.vector. (**B**) Cells were treated as described in A, and then assayed for cell death using annexin V staining, a measure of apoptosis, was determined 48 h later by fluorescence-activated cell sorting (FACS) analysis using the CellQuest software (Becton Dickinson). An average of three independent experiments is shown ± SD as errors bars, *, *p* < 0.001 comparted to Ad.vector. (**C**) MCF-7 cells were treated for 24 h with Ad.IL-24 (25, 50, and 100 pfu per cell), 10 μM H-89 or Ad.vector (100 pfu per cell). Cells were collected, protein purified, and subjected to Western blot analysis to detect phospho-p38 MAPK, total p38 MAPK, and β-actin. (**D**) MCF-7 cells were infected with either Ad.vector (control) or Ad.IL-24 at 100 pfu/cell, and either untreated or treated with 10 μM H-89 for 72 h. Western blot analysis was performed with antibodies for phospho-p38 MAPK, total p38 MAPK, and β-actin.

Next, we explored whether PKA is involved in IL-24-induced apoptosis in breast cancer cells. To test this hypothesis, we treated several human breast cancer cell lines (MCF-7, T47D, MDA-MB-157, and MDA-MB-231) with IL-24 in the presence or absence of the specific PKA inhibitors, H-89, and PKI peptide (sequence: Myr-Gly-Arg-Thr-Gly-Arg-Arg-Asn-Ala-Ile-NH_2_). Cell viability and induction of apoptosis was measured by 3-(4,5-dimethylthiazol-2-yl)-2,5-diphenyltetrazolium bromide MTT and annexin V-fluorescein isothiocyanate/propidium iodide (FITC/PI) assays, respectively. As shown in Figure 3A,B, H-89, and PKI inhibited IL-24-mediated killing in all breast cancer cell lines. Taken together, these results suggest that PKA is necessary for IL-24-induced apoptosis. We have previously demonstrated that IL-24 selectively kills cancer cells through p38 mitogen-activated protein kinase (p38 MAPK) signaling [21]. Because p38 MAPK participates in the regulation of the ER stress signaling, we hypothesized that PKA may be involved in IL-24 activation of p38 MAPK. As shown in Figure 3C, IL-24 activates p38 MAPK at threonine 180 and tyrosine 182 in MCF-7 cells. To validate that PKA plays a role in IL-24-mediated activation of p38 MAPK, we used H-89 in conjunction with IL-24 treatment and found that the inhibition of PKA prevents the activation of p38 MAPK over 24 h, suggesting that p38 MAPK signaling pathway is downstream of PKA (Figure 3D). 

### 2.4. IL-24 Activates TP53, a Downstream Target of PKA Activity

**Figure 4:**
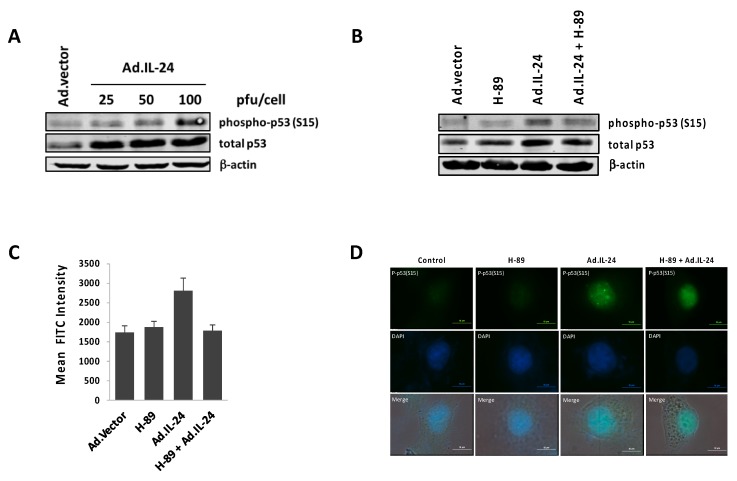
IL-24 induces TP53 expression, and promotes nuclear translocation in a PKA-dependent manner. (**A**,**B**) MCF-7 cells were treated for 72 h with Ad.IL-24 (25, 50, and 100 pfu per cell), 10 μM H-89, or Ad.vector (100 pfu per cell). Cells were collected, protein purified, and subjected to Western blot analysis to detect to detect phospho-TP53, total TP53, and β-actin proteins. (**C**) Semi-quantitative measurements of the mean fluorescein isothiocyanate (FITC) intensity were taken using Nikon NIS Elements whereby, the average intensity of five cell nuclei were taken under each experimental condition. Error bars are expressed as the standard deviation of FITC intensity values. (**D**) Cells were fixed and phospho-Ser15 TP53 was detected by immunofluorescence using anti-phospho-TP53 antibodies.

Previous studies have shown that IL-24 is able to activate TP53 in breast cancer, hepatocellular carcinoma and chronic lymphocytic leukemia cells [22,23,24]. However, until now, there is no evidence showing that IL-24 activates PKA to regulate TP53 activity in cancer cells. Here, we show that IL-24 induces TP53 expression, increases its phosphorylation on serine 15 (phospho-Ser15 TP53), and it promotes nuclear translocation in MCF-7 breast cancer cells in a PKA-dependent manner (Figure 4). It is known that the nuclear translocation of phospho-Ser15 TP53 plays a critical role in the regulation of cell cycle arrest, apoptosis, and cellular senescence in cancer cells [25,26,27,28,29,30,31,32]. Our results reveal that in response to IL-24, phospho-Ser15 TP53 translocates from the cytoplasm into the cell nucleus compared to the Ad.vector, and H-89 reduces this translocation (Figure 4C). Taken together, these results suggest that IL-24-induced TP53 expression, phosphorylation, and nuclear localization is mediated by PKA activity. 

### 2.5. PKA Activation Mediates the IL-24 Extrinsic Apoptotic Effect

**Figure 5:**
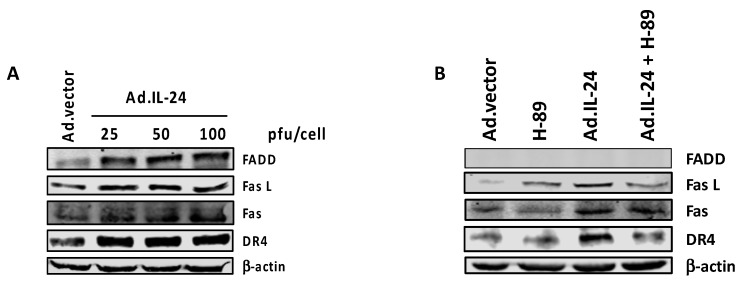
The inhibition of PKA blocks IL-24 activation of extrinsic apoptosis. (**A**) MCF-7 cells were infected with either Ad.vector (control) or increasing concentrations of Ad.IL-24 (25, 50, 100 pfu per cell) for 72 h. Western blot analysis was performed with antibodies for FasL, Fas, FADD, DR4, and β-actin. (**B**) MCF-7 cells were infected with either the Ad.vector (control) or Ad.IL-24 at 100 pfu/cell, and either untreated or treated with 10 μM H-89 for 72 h. Western blot analysis was performed with antibodies for FasL, Fas, FADD, DR4, and β-actin.

IL-24 treatment is known to induce the expression of various members of the extrinsic apoptotic pathway, such as Fas cell surface receptor (Fas), Fas ligand (FasL), Fas-associated death domain (FADD) in human ovarian cancer cells, and death receptor 4 (DR4), in colorectal cancer cell lines [33,34]. Based on these studies, we sought to determine whether IL-24 activates the extrinsic pathway of apoptosis in MCF-7 breast cancer cells, and whether the IL-24 induction of extrinsic apoptotic pathway is PKA-dependent. IL-24 infection did indeed increase Fas, FasL, DR4, and FADD levels in MCF-7 cells within 72 h (Figure 5A). The increase in the expression of the molecular markers associated with the extrinsic apoptotic pathway was blocked by H-89 (Figure 5B). Taken together, these results suggest that PKA is necessary for the IL-24-induced extrinsic apoptotic pathway. 

## 3. Discussion 

Based on the positive results from pre-clinical and Phase I clinical trials [35], IL-24 has been transitioned into a phase II clinical trial, indicating that it is has the potential to be safe and effective for cancer gene therapy. We and others have extensively investigated and reported the underlying apoptotic mechanisms of IL-24 protein treatment and Ad.IL-24 infection in preclinical studies in several cancer cells, including melanoma, glioblastoma, breast, prostate, lung, colon, liver, and cervical cancer cells [3,4,5,6,10,36,37,38]. We have shown that Ad.IL-24 and IL-24 protein, produced by Ad.IL-24-infected cells, display extensive cancer-specific pro-apoptotic activity by stimulating ER stress and ceramide production in prostate cancer cells with no effect on normal prostate epithelial cells [39]. Secreted IL-24 protein also generates a strong expression of endogenous IL-24, and subsequent induction of tumor-specific killing by ER stress activation and reactive oxygen species production [10]. IL-24 also inhibits angiogenesis in human lung tumor cells in vivo, and sensitizes breast cancer cells to chemotherapy [1,4]. More recently, we have shown that IL-24-triggered-phosphorylation of eIF2α reduces the availability of the ternary complex in squamous cell carcinoma KLN cells, and preferentially represses the expression of oncogenic proteins and increases the expression of pro-apoptotic and tumor suppressor proteins [14].

Although signaling pathways triggered by IL-24 have been the focus of intensive studies for over 20 years, the mechanisms governing cancer-specific apoptosis triggered by IL-24 are still not well understood. The results presented here identify PKA as a key mediator of cancer-specific killing by IL-24. For the first time, we show that IL-24 increases intracellular cAMP levels, and activates PKA to exert its killing effect through the extrinsic apoptotic pathway in MCF-7 breast cancer cells (Figure 5). IL-24 elevates cAMP levels in a concentration-dependent manner, resulting in PKA activation, and increases the phosphorylation of PKA substrates (Figure 2A,B) Cell viability and annexin V analysis shows that cancer cell killing by IL-24 is dependent on PKA, as chemical inhibition of PKA by H-89 abrogates the pro-apoptotic activity of IL-24 on cancer cells (Figure 3A,B). In addition, PKA plays a role in IL-24-mediated ER stress, as evidenced by activation of ATF4 (Figure 2D) 

We observe for the first time that in response to IL-24 treatment, PKA plays a role in the extrinsic apoptotic pathway. When MCF-7 breast cancer cells are treated with IL-24, key players in the Fas signaling pathway, such as Fas, FasL, and FADD, are upregulated, supporting previous studies that IL-24 activates apoptosis extrinsically [33,34,40]. With the addition of the PKA inhibitor H-89, these apoptotic signaling proteins are downregulated, indicating that the induction of extrinsic apoptotic pathways by IL-24 is mediated by PKA (Figure 5B). 

In human cancer, tumor suppressor TP53 possesses high mutation rates, and its inhibition or absence is critical in driving cancer formation and progression. We reveal for the first time that PKA activation is necessary for IL-24 activation of TP53 at serine 15 (phospho-Ser15 TP53) (Figure 4B). Phospho-Ser15 TP53 is known to localize to the nucleus to activate cell-cycle arrest and apoptosis in cancer cells [25,26,27]. Here, we provide evidence that phospho-Ser15 TP53 localizes to the nucleus after IL-24 treatment in MCF-7 cells (Figure 4C). This localization to the nucleus is also dependent on the activity of PKA. Earlier studies have established that IL-24 activates TP53 in MDA-MB-453 breast cancer cells and HepG2, MHCC97L and Hep3B hepatocellular carcinoma cells [22,23]. As shown in Figure 2, IL-24 inhibits the growth of different breast cancer cells lines containing wild-type TP53 (MCF-7), mutant TP53 (MDA-MB-231 and T47D), or null TP53 (MDA-MB-157). While we have shown that IL-24 can induce apoptosis independent of TP53, the molecular mechanisms underlying the activation of TP53 triggered by IL-24 could be relevant for the development of precise therapeutics that use IL-24 in combination with compounds that reactivate mutant TP53 to wild-type TP53 to suppress tumors (Reviewed in [41]). These small-molecule drugs, which have been tested in pre-clinical and Phase I trials, can specifically target mutant forms of TP53 to reactivate its structural stability, or restore its transcriptional activity. Future studies examining the synergistic effects of IL-24 and these compounds can uncover new treatment options for patients that have tumors with mutated TP53 gene profiles. 

## 4. Materials and Methods

### 4.1. Cells Culture and Reagents

Human breast MCF-7, MDA-MB-231, MDA-MB-157, and T47D cell lines were acquired from American Type Culture Collection (ATCC), maintained per ATCC protocols and utilized within six months of thawing. Cell lines were grown in a humidified atmosphere at 37 °C with 5% CO_2_, and culture media was replaced every other day. Dulbecco’s Modified Eagle’s medium (DMEM) was used. H-89 [*N*-(2-aminoethyl)-5-isoquinolino-sulfonamide] was purchased from EMD Millipore (Darmstadt, Germany). PKI Myristoylated Peptide (Sequence: Myr-Gly-Arg-Thr-Gly-Arg-Arg-Asn-Ala-Ile-NH_2_) was purchased from Invitrogen (Camarillo, CA, USA). 

### 4.2. Virus Infection

After attachment within 24 h, cells were infected with the IL-24 expressing replication defective adenovirus (Ad.IL-24) or the control, a corresponding empty adenovirus vector lacking exogenous genes (Ad.vector). The adenoviruses were custom made by Vector Biolabs, Inc. (Philadelphia, PA, USA).

### 4.3. MTT Assays

Prior to treatment, cells were plated at a concentration of 2 × 10^3^ cells/well in 96-well dishes. Cells were grown in DMEM with 10% FBS (Gibco, Waltham, MA, USA) and attached 12 h prior to treatment(s). After Ad.IL-24 treatment, cells were treated with inhibitors and media was replaced at day 3 with fresh inhibitor. After five days of treatment, cell proliferation and viability were determined by 3-(4,5-dimethylthiazol-2-yl)-2,5-diphenyltetrazolium bromide (MTT) staining (Sigma, St. Louis, MO, USA), as previously described in [42]. Absorbance was measured at 595 nm using Tecan Spark 10 M Microplate Reader (Männedorf, Switzerland) and is proportional the number of living cells present.

### 4.4. Annexin V Binding Assays

After treatment, cells were detached using trypsin and washed once with complete DMEM medium and phosphate-buffered saline (PBS). Cells were then re-suspended in 500 μL of binding buffer containing 2.5 mmol/L CaCl_2_, and stained with allophycocyanin-labeled annexin V (Becton Dickinson Biosciences, Palo Alto, CA, USA) and propidium iodide (PI) at room temperature for 15 min. Immediately after staining, flow cytometry was performed. Flow cytometry assays were performed as previously described [14].

### 4.5. Western Blot Analysis

Cellular protein was extracted using Pierce IP Lysis Buffer (Thermo Scientific, Rockford, IL, USA) and a mixture of Halt Protease Inhibitor Cocktail 100X (Thermo Scientific, Rockford, IL, USA) and Phosphatase Inhibitor Cocktail 100× (Cell Signaling Technology, Danvers, MA, USA). Sixty micrograms of protein were applied to a 10% SDS/PAGE and transferred to nitrocellulose membranes. Membranes were incubated with Odyssey blocking buffer (LI-COR Biosciences, Lincoln, NE, USA) prior to incubation with polyclonal or monoclonal antibodies to phospho-PKA substrates, PKA-Cα subunit, FasL, Fas, FADD, DR4, phospho-S15 TP53, total TP53, phospho-p38 MAPK, total p38 MAPK, phospho-S245 ATF4, total ATF4, BiP, and β-actin overnight at 4 °C. All primary antibodies were purchased from Cell Signaling Technology (Danvers, MA, USA) with the exception of phospho-S245 ATF4 from Sigma Aldrich (St. Louis, MO, USA). Goat anti-rabbit IgG (H + L) 800 CW, goat anti-rabbit (680 RD) and/or goat anti-mouse (H + L) secondary antibodies were applied for 1 h at room temperature (1:25,000, LI-COR) prior to washing with 1× Tris Buffered Saline Tween-20 (TBS-T). Visualization was carried out with the LI-COR Odyssey CLx imaging system and software. 

### 4.6. cAMP Assay

MCF-7 cells were plated at a concentration of 5 × 10^3^ cells/well in Biocoat Poly-D-Lysine 96-well dishes. Cells were grown in DMEM with 10% FBS and attached 24 h prior to treatments. Production of cyclic adenosine monophosphate (cAMP) was determined by the cAMP-Glo^TM^ Assay by Promega (Madison, WI, USA). Luminescence was measured using a Tecan Spark 10M Microplate Reader (Männedorf, Switzerland).

### 4.7. Immunofluorescence

Cells were plated onto chamber slides (Falcon; BD Biosciences, San Jose, CA, USA) and maintained per ATCC protocols. After 24 h of Ad.IL-24 infection and H-89 treatment, cells were fixed with 2% paraformaldehyde, permeabilized by 0.1% Triton X-100, and then incubated with phospho-Ser15 TP53 antibody. Incubation buffer was added to chamber slides as negative control. After 2 washes with wash buffer, chamber slides were then incubated with Alexa Fluor 594 secondary antibody (Jackson Laboratory, Bar Harbor, ME, USA) for 1 h at room temperature. After rinsing with wash buffer, chamber slides were coated with 4′,6-diamidino-2-phenylindole (DAPI) anti-fade mounting medium (Life Technologies, Grand Island, NY, USA). DAPI-stained nuclei, cells were assessed on an inverted fluorescent microscope (Nikon EclipseTi, Melville, NY, USA) using 400× total magnification. Semi-quantitative measurements of the mean FITC intensity of cell nuclei were taken using NIS Elements software, whereby the average intensity of five cells were taken under each experimental condition. 

### 4.8. Statistical Analysis

Experiments were performed at least in duplicate or triplicate, and data represent the average of three independent experiments. Statistical analysis was performed using Student *t* test. *p* values less than or equal to 0.05 were considered significant. Experiments shown are the means of multiple individual points from multiple experiments (±SEM).

## 5. Conclusions

**Figure 6:**
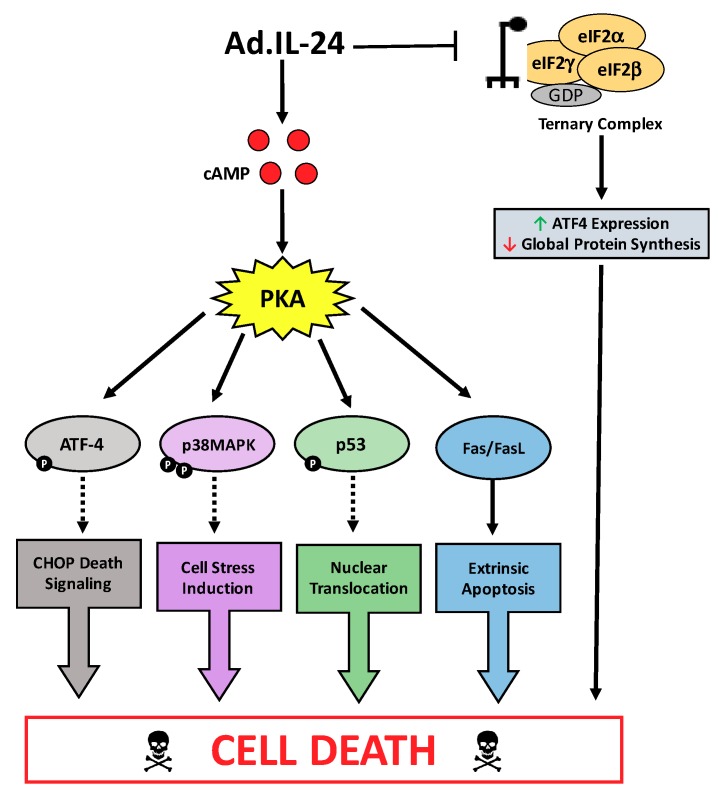
IL-24 activates PKA to induce apoptosis in breast cancer cells. A schematic of the molecular mechanisms underlying Ad.IL-24 induced apoptosis of cancer cells, involving PKA activation, ATF-4 phosphorylation, p38MAPK signaling, p53 phosphorylation, Fas apoptotic signaling, and the inhibition of translation initiation (red arrow indicates downregulation; green arrow indicates upregulation; black arrows indicate pathway activation, black dotted arrows indicate potential mechanism of action, black bar headed arrow indicates inhibition).

Overall, these results demonstrate for the first time that PKA is a key mediator of IL-24-induced apoptosis in breast cancer cells. Specifically, we reveal that PKA mediates several pathways, including p38 MAPK, p53, ER stress, and extrinsic apoptosis, which are known to be activated by IL-24 to specifically kill cancer cells (Figure 6). The evidence presented here points to PKA as a possible upstream regulator that potentiates IL-24 killing of cancer cells. Understanding the complexities of IL-24 induction of apoptosis in cancer cells significantly broadens its potential as an anti-tumor therapeutic and reveal new combinatorial strategies for targeted cancer therapies.
